# The progression pattern of male hyrax songs and the role of climactic ending

**DOI:** 10.1038/s41598-017-03035-x

**Published:** 2017-06-05

**Authors:** Vlad Demartsev, Amiyaal Ilany, Arik Kershenbaum, Yair Geva, Ori Margalit, Inbar Schnitzer, Adi Barocas, Einat Bar-Ziv, Lee Koren, Eli Geffen

**Affiliations:** 1 Dept. of Zoology, Tel Aviv University, Tel Aviv, 69978 Israel; 2 Dept. of Evolutionary Biology and Environmental Studies, University of Zurich, Zurich, 8057 Switzerland; 3 The Mina and Everard Goodman Faculty of Life Sciences, Bar-Ilan University, Ramat-Gan, 52900 Israel; 4 Dept. of Zoology, University of Cambridge, Cambridge, UK; 5 Dept. of Zoology and Physiology and Program in Ecology, University of Wyoming, Laramie, WY 82071 USA; 6 Mitrani Dept. of Desert Ecology, Ben-Gurion University of the Negev, Be’er Sheva, 8499000 Israel

## Abstract

The study of animal vocal signals can either focus on the properties of distinct vocal elements or address the signal as a whole. Although some attention has been given to the continuous progression patterns of bird songs, such patterns in mammalian vocalisations have been largely overlooked. We examined temporal changes in structural and acoustic parameters in male rock hyrax songs. We found a gradual increase in call frequency and amplitude towards the song ending, as well as an abrupt increase in bout syntactic complexity, peaking in the last quintile of a song. In musical terms, such a pattern can be described as a crescendo (amplitude increase) with a terminal climax. In Western music, crescendos are used to maintain attention and direct the listeners towards a memorable highpoint of the musical piece. This structure may have an analogous function in animal communication, recruiting audience attention towards the climactic and potentially most informative part of the signal. Our playback experiments revealed that hyrax males tend to reply more to songs with a climactic ending, indicating that this progression pattern is important for hyrax communication. We suggest that animal vocal communication research can benefit from adding musical concepts to the analysis toolbox.

## Introduction

Although animal acoustic signals often form long and complex sequences^1^, traditionally most of the research on animal song structure has focused on classifying the signal into distinct units (syllables). This allowed easy quantitative analysis of repertoire size, singing versatility and specific component production^1, 2^. However, such analysis can only reveal the first-order features of the signal and does not always account for such aspects as signal progression and dynamic structure, which might also bear information and influence the listeners. Dynamic features, such as formation of phrases containing a typical beginning, middle and end, stereotypic rhythms and pitch intervals, might bind seemingly discrete song elements into a single cohesive percept. Although there is an increasing interest in such signal properties and several attempts have been made to assess the continuous parameters of vocalisation^3–6^, this approach is still far from being a common practice in animal vocalisation research.

Human music is able to induce emotional reactions and affect the behaviour of listeners^7^. Crescendo - a gradual increase in amplitude or intensity in a musical piece^8^, has been found to create an arousal effect in listeners^9^. An ending of a musical piece often consists of dynamic rhythmic changes or/and an amplitudinal climax, possibly to capture the audience’s attention. The entire musical piece preceding the dramatic ending may funnel the listener towards that single highpoint at the end^10^, by establishing sender-receiver contact and creating expectations. Repetitions and innovations during the introduction build suspense and maintain listener attention^11^ towards the powerful ending^10^.

Applying musical concepts to animal call features might reveal similar aspects of their effects on the behavioural states of listeners^2^. The term “zoomusicology” established an analogy between non-human calls and human music several decades ago^12^. The subject has mostly received attention in birds, after Charles Hartshorne^13^ suggested that every aspect of human music exists in bird songs: e.g. accelerando (gradual increase in tempo) in the field sparrow (*Spizella pusilla*) and ruffed grouse (*Bonasa umbellus*); ritardando (gradual decrease in tempo) in the yellow-billed cuckoo (*Coccyzus americanus*); crescendo (gradual increase in loudness) in Heuglin’s robin (*Cossypha heuglini*) and diminuendo (gradual decrease in loudness) in the Misto yellowfinch (*Sicalis luteola*)^2^. In addition to the descriptive notion of musical features in animal calls, several attempts have been made to suggest a functional explanation for the phenomenon. For example, in the thrush nightingale (*Luscinia luscinia*) a song’s temporal structure resembling an accelerando-like rhythm acceleration was found to progress through several song phrases towards a glissando (high-pitch sweep) finish. This gradual build-up towards culmination, manipulation of rhythmic timing and amplitude, could function to maintain the attention of receivers^2^ and avoid signal habituation^14^.

Several mammals also produce songs with features similar to those used in music. Most famously, humpback whales (*Megaptera novaeangliae*) produce hours-long structured songs that involve highly styled repetitions of phrases and motifs^15^. Harbour seals (*Phoca vitulina*) produce long and complex vocal displays that, despite their inharmonious nature, have been classified as songs^16^. Among primates, the indri (*Indri*), tarsiers (*Tarsius*), titi monkeys (*Callicebus*) and gibbons (*Hylobates*) are known to sing. Siamang (*Hylabates syndactylus*) mated pairs produce long and stereotyped duets that include multiple different phrases and male-female transitions^17^.

In this work, we applied musical terminology to describe progression patterns of rock hyrax (*Procavia capensis*) vocalisation and discuss the theoretical parallelism between the functional effects of those patterns on the audience in both music and animal calls. In addition, we experimentally examined whether the climactic ending in hyrax songs has a communicational significance and affect the behaviour of conspecific listeners.

**Figure 1:**
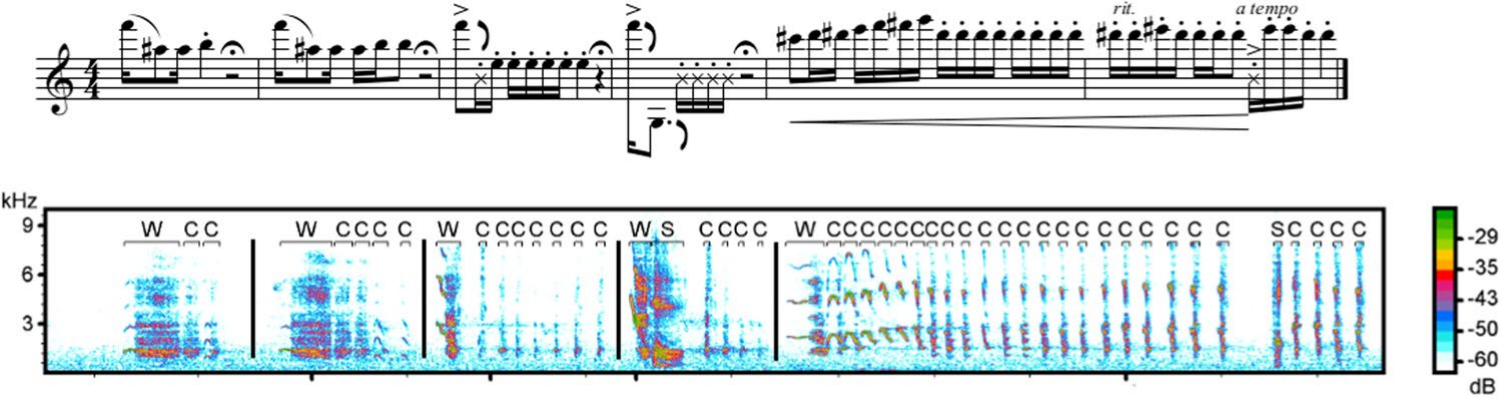
(**a**) Proposed musical notation of male song corresponding to the parts represented in the spectrogram. (**b**) Sample spectrogram of male rock hyrax song. Different vocal elements are marked and coded as **W**-Wail, **C**-Chuck and **S**-Snort. The representation order of singing bouts corresponds to natural temporal progression of a typical male hyrax song (beginning – low complexity, middle – low/intermediate complexity. end – high complexity). Vertical lines mark bout boundaries.

Adult rock hyrax males can be clearly identified as “bachelors”, who are mostly solitary and do not associate with any group^18^, and “residents” who reside with a group of females, routinely interact with them and are observed mate-guarding older and experienced females^19^. Both bachelors and residents produce long, complex vocalisations (songs), increasing in frequency towards the mating season. Male songs can be “spontaneous” (performed without any observable external trigger) or “induced” (performed following a specific external event; e.g. predator presence, conspecific alarm or distress calls, agonistic interaction, etc.)^20^. Hyrax male songs consist of three vocal elements (wail, chuck and snort; Fig. 1) arranged in multiple bouts separated by distinct silent intervals^21, 22^, and are audibly recognized as progressively increasing in intensity (Fig. 1). Male calls (songs; Fig. 1) have been shown to reflect caller identity, age, social rank and body condition^22–24^. Receivers are sensitive to both song structure^25^ and to the signallers’ identity and individual traits^26^. Signallers are attuned to the state of their audience and attempt to time their performance to periods of increased listener attention^27^. Moreover, males increase the overall rhythm and complexity of their songs in the presence of an alert audience^20^. Until now, the analysis of hyrax songs has been based on discrete measures of spectral parameters or on the composition of the different vocal elements within a song.

Here, we analysed hyrax songs from a different perspective. By viewing each song as a single unit, we addressed the song syntactic complexity and acoustic parameters on a wider scale that accounted for the dynamics of the signal progression. Demartsev, *et al*.^20^ suggested that the syntactic complexity of a song might reflect the signallers’ quality and play a role in male self-advertisement. We thus hypothesized that the complexity progression pattern would be more pronounced in the signals of high-quality males. Furthermore, songs that deviate from the general progression pattern might be less attractive to the audience and/or transmit signals of lower quality. We predicted that such songs would receive fewer replies from conspecific receivers in comparison to songs that reach a climactic ending.

## Results

### Natural song progression analysis

We analysed 140 previously recorded songs under natural conditions, performed by 24 adult males, out of 188 songs recorded^20^. Forty eight songs, which were less than eight bouts long, were omitted from the analysis. Since the mean number of bouts/song (±SD) was 22.9 ± 19.7 bouts, we suspected that extremely short songs might have been terminated prematurely and were less suitable for the analysis of progression. Out of the 140 songs used, 60% showed an ascending progression pattern towards the song ending, 30.7% of songs did not show significant change, and 9.3% of songs showed a significant decrease in bout duration and/or entropy rate towards the song ending.

**Figure 2:**
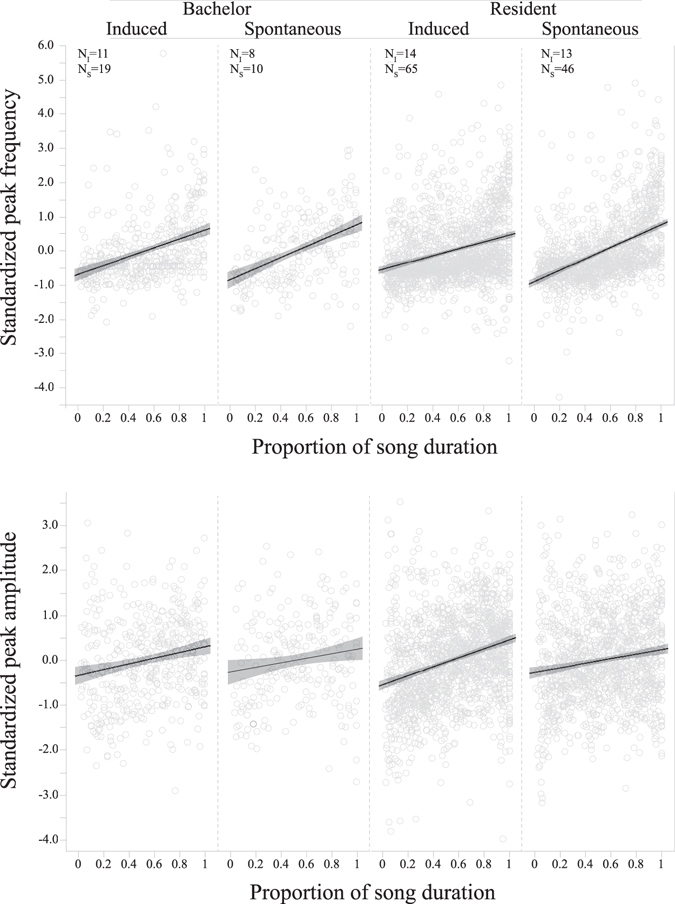
Progression plots of (**a**) standardised peak frequency and (**b**) standardised peak amplitude of vocal element and their proportional location through the signal. The trend lines show linear slope estimates with CI as shaded areas. **Bachelor** songs were performed by males without stable association with a female group. **Resident** songs were performed by males that were regularly observed associating with a female group. **Induced** songs were performed following an external trigger event. **Spontaneous** songs were performed without any observable trigger. **N**_**i**_ denotes number of individual males and **N**_**s**_ denotes number of songs in a corresponding category.

**Figure 3:**
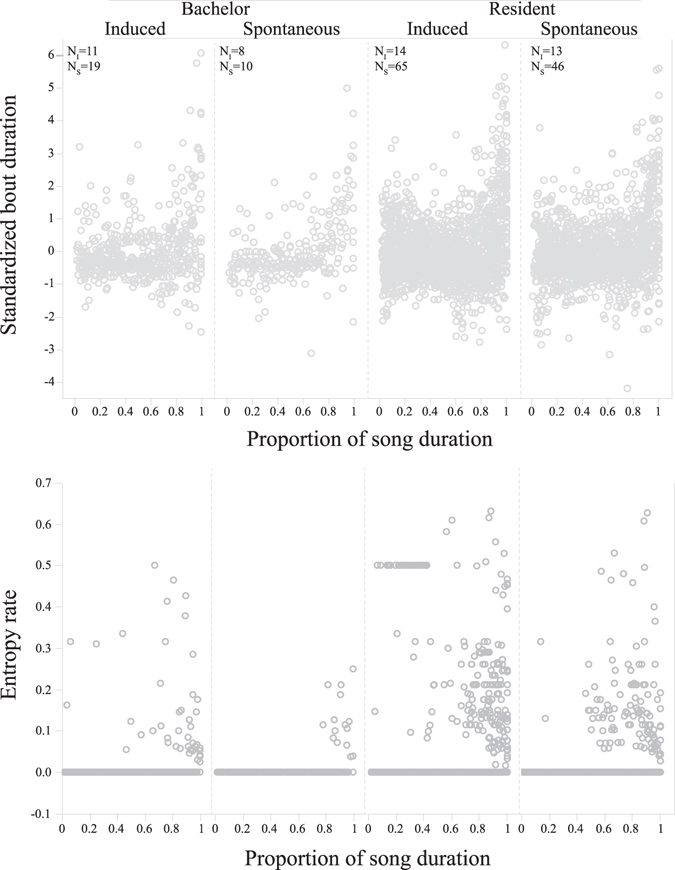
Progression plots of (**a**) standardised bout duration and (**b**) bout entropy rate and their proportional location through the signal. **Bachelor** songs were performed by males without stable association with a female group. **Resident** songs were performed by males that were regularly observed associating with a female group. **Induced** songs were performed following an external trigger event. **Spontaneous** songs were performed without any observable trigger. **N**_**i**_ denotes number of individual males; **N**_**s**_ denotes number of songs in a corresponding category.

To test for progression in male hyrax songs, we used mixed models where male residency (resident or bachelor) and singing context (spontaneous or induced) were set as fixed effects, the relative temporal position of each vocal element in the song (hereafter proportion of song duration) was set as continuous predictor (covariate), the 2-way interactions between fixed effects and proportion of song duration was included in the model, and male identity was set as a random effect. We tested the effect of the above explanatory variables on three vocal parameters of the chuck and wail elements (Table 1; Fig. 2) and on four bout characteristics in the male song (Table 2, Fig. 3).

**Table 1:** The effect of male residency (M_RS_; resident or bachelor), singing context (M_SC_; spontaneous or induced) and song progression (proportion of song duration; M_SP_) on peak amplitude per bout, peak frequency per bout, and fundamental frequency per bout in the wail and chuck elements of hyrax male songs.

Term	Estimate	Wald χ^2^	P	Term	Estimate	Wald χ^2^	P
***Wail***				***Chuck***			
*Peak amplitude*							
M_RS_	0.13	0.4	0.531	M_RS_	0.13	0.4	0.545
M_SC_	−0.05	0.1	0.770	M_SC_	−0.23	2.2	0.139
M_SP_	0.88	11.4	**0**.**001**	M_SP_	0.54	8.6	**0**.**003**
M_RS_ * M_SP_	−0.27	0.4	0.524	M_RS_ * M_SP_	−0.23	0.3	0.560
M_SC_ * M_SP_	0.12	0.1	0.716	M_SC_ * M_SP_	0.42	2.1	0.151
*Peak frequency*							
M_RS_	−0.08	0.3	0.586	M_RS_	−0.07	0.1	0.732
M_SC_	0.15	4.1	**0**.**044**	M_SC_	0.32	5.2	**0**.**022**
M_SP_	1.67	99.1	**<0**.**001**	M_SP_	1.61	56.7	**<0**.**001**
M_RS_ * M_SP_	0.14	0.2	0.664	M_RS_ * M_SP_	0.16	0.2	0.681
M_SC_ * M_SP_	−0.27	3.1	0.079	M_SC_ * M_SP_	−0.60	5.6	**0**.**018**
*Fundamental frequency*							
M_RS_	0.16	1.0	0.316	M_RS_	−0.02	0.0	0.932
M_SC_	0.22	2.5	0.112	M_SC_	0.05	0.1	0.762
M_SP_	1.32	40.5	**<0**.**001**	M_SP_	0.89	35.7	**<0**.**001**
M_RS_ * M_SP_	−0.33	1.0	0.310	M_RS_ * M_SP_	0.04	0.0	0.891
M_SC_ * M_SP_	−0.42	2.2	0.139	M_SC_ * M_SP_	−0.10	0.1	0.734

*df* = 1 in all effect tests.

**Table 2:** The effect of male residency (M_RS_; resident or bachelor), singing context (M_SC_; spontaneous or induced) and song progression (proportion of song duration; M_SP_) on bout duration, the number of chucks and snorts per bout, and bout entropy rate in hyrax male songs.

Term	Slope	Wald χ^2^	P
*Bout duration*			
M_RS_	−0.17	1.1	0.289
M_SC_	0.17	2.7	0.099
M_SP_	0.99	43.9	**<0**.**001**
M_RS_ * M_SP_	0.32	1.1	0.298
M_SC_ * M_SP_	−0.32	2.7	0.098
*Number of chucks*/*bout*			
M_RS_	0.10	0.5	0.498
M_SC_	0.17	2.9	0.091
M_SP_	2.30	250.8	**<0**.**001**
M_RS_ * M_SP_	−0.20	0.5	0.474
M_SC_ * M_SP_	−0.33	2.9	0.088
*Number of snorts*/*bout*			
M_RS_	−0.05	0.3	0.566
M_SC_	−0.12	1.4	0.241
M_SP_	1.29	215.6	**<0**.**001**
M_RS_ * M_SP_	0.09	0.3	0.574
M_SC_ * M_SP_	0.22	1.4	0.243
*Bout entropy rate*			
M_RS_	−0.00	0.1	0.742
M_SC_	0.01	0. 3	0.595
M_SP_	0.43	35.3	**<0**.**001**
M_RS_ * M_SP_	−0.01	0.4	0.523
M_SC_ * M_SP_	0.00	0.0	0.861

*df* = 1 in all effect tests.

A significant positive slope in the proportion of song duration (M_sp_; Tables 1, 2) indicated an increase in the dependent variable along the song. Peak frequency, peak amplitude and fundamental frequency of elements all showed a clear trend of gradual increase during song progression (Table 1, Fig. 2). Except for peak frequency, this progression pattern was similar in both induced and spontaneous songs performed by males of both residency categories (Table 1). The increase in peak frequency was significantly different between spontaneous and induced songs for both the wail and the chuck elements (Table 1). Peak frequency in the wail element was higher in induced songs compared to spontaneous songs (P = 0.044; Table 1) but in both song types peak frequency increased along the song at equal rate (Table 1). For the chuck element, we detected a significant interaction between singing context and proportion of song duration (P = 0.018; Table 1). Peak frequency increased along the song at higher rate in spontaneous songs (GEE, slope = 1.64, Wald χ^2^ = 114.5, *df* = 1, P < 0.001) compare with induced songs (GEE, slope = 1.05, Wald χ^2^ = 19.2, *df* = 1, P < 0.001). In contrast to the gradual increase in frequencies, bout duration, number of chucks and snorts per bout, and entropy rate all showed a significant abrupt increase towards the end of the song, whereas the initial 50–80% of the song was maintained relatively constant (Fig. 3). This increase in bout complexity along the song was independent of male residency or singing context as both of these fixed effects and their interaction were insignificant in all tests (Table 2).

### Playback analysis

**Figure 4:**
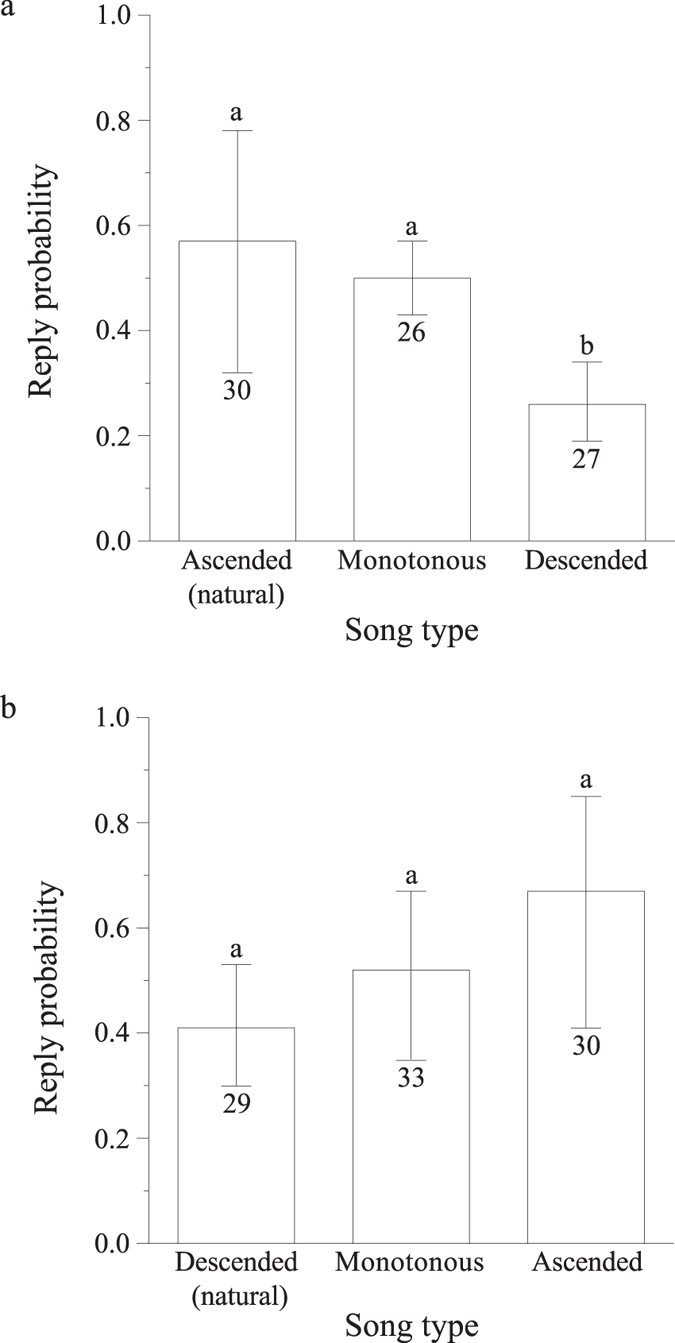
Reply rates (±95% Wald Confidence Intervals) to playback experiments. (**a**) SetA (descending playbacks) – reply rates to natural, ascending control songs (N_ASC_) and synthetic monotonous (S_MON_) and synthetic descending (S_DSC_) versions. (**b**) SetB (ascending playbacks) – reply rates to natural, descending control songs (N_DSC_) and synthetic monotonous (S_MON_) and synthetic ascending (S_ASC_) versions. Letters above error bars denote statistical significance (P < 0.05) and values below error bars denote number of playbacks.

In Set A we compared the rate of reply between playbacks of natural ascending songs (i.e. control; unaltered songs with increase in entropy and bout length) to playbacks of synthetic monotonous or descending songs. Playback type was set as the fixed effect and social group identity and track number as the random effects. The reply rate was significantly different between treatments (GEE, Wald χ^2^ = 34.9, *df* = 2, P < 0.001, n = 83). The synthetic monotonous songs, which lacked the increase in bout length and bout entropy towards the end, were replied to at a similar rate to that of natural songs (control, multiple comparisons by sequential Bonferroni P = 0.568). However, the reply rate to synthetic descending songs, with decreased bout length and entropy at the final stages of the song, was significantly lower, showing only ~30% probability of reply in comparison to ~50% in naturally ascending (P = 0.007) and synthetic monotonous (P < 0.001) songs (Fig. 4a).

The complementary Set B trials revealed a similar trend of natural descending songs receiving the lowest reply rate (~40%) and synthetic ascending songs receiving the highest reply rate (~70%). Synthetic monotonic songs received an intermediate ~50% reply rate (Fig. 4b). Although this trend of linear increase in reply rate with the increase in bout duration and bout entropy was strongly pronounced, it was not statistically significant (GEE, Wald χ^2^ = 2.1, *df* = 2, P = 0.351, n = 92).

To accommodate the effect of both male and track identities in the trials on reply rate, we repeated the above analysis after setting both male and track identities as random effects. The reply rate between the treatments of set A was significantly different (GEE, Wald χ^2^ = 7.7, *df* = 2, P = 0.021, n = 83). The reply rate to the synthetic descending songs was significantly lower than to the natural songs (P = 0.016). The reply rate between the treatments of set B was insignificant as above (GEE, Wald χ^2^ = 2.7, *df* = 2, P = 0.254, n = 92).

Further, we tested all 24 different (natural and synthetic) playback tracks for the effect of bout duration and bout entropy rate on the conspecific reply rate. We used the regression slope between the proportion of song duration and bout duration (Fig. S1) or bout entropy rate as a predictor for reply rate. In this model, both regression slopes were the independent variables, reply rate was the dependent, and both male and track identities were set as the random effects. The reply rate significantly increased with the rise in the slope of bout entropy rate (GEE, Wald χ^2^ = 5.1, *df* = 1, P = 0.023, n = 175). A similar trend, although insignificant, was observed for bout duration (Wald χ^2^ = 2.9, *df* = 1, P = 0.087, n = 175). The effect of the interaction between bout entropy rate and bout duration on the reply rate was insignificant (Wald χ^2^ = 1.0, *df* = 1, P = 0.321). These results suggest that males tend to reply significantly more to songs that end up with higher complexity.

Last, in order to control for possible effects of audio track manipulation, we have performed a pairwise comparison of reply rate between natural ascending and synthetic ascending tracks, and a complementary comparison of reply rate between natural descending and synthetic descending tracks. No significant difference in reply rate was found between the natural and synthetic ascending (GEE, Wald χ^2^ = 0.453, *df* = 1, P = 0.501, n = 60) or descending (Wald χ^2^ = 1.9, *df* = 1, P = 0.167, n = 56) tracks.

## Discussion

Our analysis of natural song progression revealed an increase in all the measured parameters throughout the signal. However, the increase rates were not identical. Peak frequency, peak amplitude and fundamental frequency increased gradually, whereas bout duration and entropy rate remained constant throughout most of the song duration, until a steep increase at the final part of the signal. Our playback experiments verified that the pattern of increased bout length and complexity towards a songs’ ending is significant for hyrax communication, as the receivers’ reply rate was higher in response to songs with a more complex ending in both experimental sets.

The functional importance of vocal temporal parameters has been shown in several mammalian species. Gibbon males produce a progressively monotonic call sequence in order to demonstrate their level of readiness and to serve as an invitation for a female to join in a duet^28^. However, in the presence of a predator, gibbons perform a “crescendo”, starting with soft notes that rapidly become louder^29^. The progression pattern across hyrax songs might be aimed at drawing audience attention, which is not easily gained, by generating expectation of a climactic signal ending. Males often initiate singing following external events that promote alertness among neighbouring individuals and exploit this heightened alertness to deliver the signal to a wider and more attentive audience^20^. In a similar manner, the gradual increase in hyrax song amplitude may attract listeners and maintain their attention. In other systems, higher amplitude signals have been found to draw more attention^30^ and to be robust to habituation or withdrawal response^31^. For example, male mice (*Mus muscus*) perform an ultrasonic loud, modulated, and syntactically complex “calling song” when they are exposed to the scent of a female, in order to attract her closer. However, when the female is already present and there is no more need to attract her attention, males switch to a less energetic song with consistent pitch and more stereotyped structure^32^.

Hyrax songs have a long crescendo build-up with a gradual increase in fundamental frequency that may be similar to an “upwards glissando” (a gradual increase in pitch)^8^. In this work, we did not experimentally assess the effects of song frequency and song amplitude on conspecific attention and reply rate. A potential follow-up work, focused on manipulating these parameters, would lead to a better understanding of their function in the context of hyrax songs. At this stage, we can only hypothesize that a crescendo structure with a gradual upward frequency change may attract a larger audience and maintain higher attentiveness towards the end of the song.

Additionally, an acoustic climax in animal calls can be associated with an elevated vocal effort that is sensitive to fatigue, aging and disease^33^. As such, the terminal call climax can serve as an honest signal of the performers’ condition near the point of exertion, consistent with the handicap principle^34^. Such “on the edge”^33^ performance might be a burden for low-quality individuals^35^, which may become exhausted after the long introductory stage and produce a less complex ending. In this case, the long warm-up may serve as a handicap to emphasize the performers’ ability. Our results, however, showed no differentiation between resident and bachelor males, as both male categories are able to produce songs with a complex ending. Singing behaviour is generally cheap for male hyraxes, at least in terms of energetic investment^36^; however, the relative cost might be higher for individuals in poor condition.

The results of our playback experiments demonstrate that the listeners can detect the climactic ending of the songs, as conspecifics tended to reply more to climactic songs in comparison to their descending variants (Fig. 4). This supports our notion of a complex ending advertising higher quality and/or drawing more attention, as higher intensity signals are often followed by an increase in the response rate^37–39^, although opposite patterns have also been reported^40, 41^.

The gain of audience attention and the handicap principle are not mutually exclusive explanations for the signal progression pattern that we observed in hyrax songs. In fact, the song’s progression pattern possibly achieves both goals: keeping the audience attentive until the end by increasing amplitude and demonstrating singer capabilities (i.e. the handicap principle) by performing a complex ending. We can find a similar pattern in music, especially in opera performances and rock music. In many opera sessions and rock songs, a gradual build-up phase is followed by a climactic finale, which involves singing performance at the edge of human vocal capabilities and extremely difficult electric guitar solos, respectively. Such a dramatic ending of a musical piece creates an exciting and memorable moment for the listeners and at the same time demonstrates the quality of the performing artists.

The parallelism between features of animal calls and human music deserves serious consideration when analysing sequential animal calls from the perspective of their communicational value. An analogy between animal and human songs has repeatedly been suggested^42–44^. Indeed, it does seem that some of the principles that govern musical structures and make it attractive and interesting to human listeners might have a similar function in non-human vocal communication. As demonstrated in this work, assessment of the signal as a whole has the potential to reveal novel information channels that can be easily overlooked by analysis that focuses mainly on distinct units. The use of musical concepts in this context could aid in explaining the potential communicative significance of such novel channels. In addition, different signal units may in fact be perceptually bound together^1^. Thus, treating the signal as a whole may be more informative and enhance our understanding of the way signals are experienced by conspecific receivers.

## Materials and Methods

### Ethical statement

This study was conducted under permits from the Israeli Nature and Parks Authority (NPA), which is the government agency responsible for supervising all wildlife research in Israel. All field procedures were in accordance with NPA guidelines and regulations for trapping, sampling and handling wild hyraxes, as well as for conducting playback. Yearly reports detailing all performed procedures and experiments involving animal subjects were submitted to NPA for assessment. A consecutive permit was granted following the approval of all performed activities by NPAs’ Permit Department. Annual permit numbers are (2002/14674, 2003/14674, 2004/17687, 2005/17687, 2007/27210, 2008/31138, 2009/32871, 2010/37520, 2011/38061, 2012/38400, 2013/38803, 2014/40185, 2015/40768 and 2016/41174). Throughout the entire course of our study, no long-term stress or interference effects were detected in the individual animals or in the population. Both the general population size and the integrity of the specific social groups in the research area remained stable.

### Field protocol

The study was conducted at the Ein Gedi Nature Reserve in Israel (31°28′N, 35°24′E) as part of a long-term project. The data for the current study were collected continuously from 2002 to 2016.

Field procedures followed previously published protocols^18, 22, 23, 36^. Briefly, hyraxes were observed during the morning activity hours, about 4 hours daily, using 10X42 binoculars (Monarch, Nikon) and a telescope with up to X75 zoom magnification (Fieldscope ED82, Nikon). Rock hyraxes were trapped using live box traps (Tomahawk Live Trap Co, Tomahawk, WI, USA) baited with cabbage and kohlrabi. The traps were set at dawn, inspected after 3–4 hours, and locked open until the next trapping session. Trapped animals were anaesthetised by intramuscular injection of ketamine hydrochloride (0.1 ml/kg). Each hyrax was individually marked with a subcutaneous transponder (DataMars SA) and either an ear tag (~0.25 grams per tag) or a light numbered collar (~5 grams). Captured hyraxes were weighed and measured. Following anaesthesia recovery (at least 120 min), the animals were released back at their capture sites and resumed full normal activity. All treatments were performed in the shade to avoid overheating.

### Residency status

Each year, male hyraxes’ residency status (i.e. bachelor or resident) was determined according to the social network algorithms described in Barocas, *et al*.^18^. Resident males were observed in a stable association with a group of females, sharing sleeping dens and feeding sites. Bachelor males showed no stable association with other individuals and were observed only in brief interactions with females during the mating season.

### Vocalisation recording and analysis

Hyrax long-range vocalisations were recorded from a distance of 10–50 m with a Sennheiser ME 67 shotgun microphone (frequency response 50–20,000 Hz ± 2.5 dB) powered by a Sennheiser K6 module, and covered with a Sennheiser MZW70-1 blimp windscreen (Sennheiser Electronic GmbH & Co. K. G., Wedemark, Germany). The microphone was hand-held using an MZS20-1 shock-mount with a pistol grip. Vocalisations were recorded in mono (Tascam HD-P2 digital audio recorder; TASCAM Corporation, Montebello, CA, USA), with a sampling frequency of 48 kHz and a sampling width of 24 bits^27^.

We analysed 140 previously recorded songs performed under natural conditions by 24 adult males. Songs were classified using the performer’s residence status (bachelor or resident) for the relevant year. For each male category, we then classified songs into two states: “spontaneous” or “induced”^20^.

For each song, a spectrogram was generated using Avisoft SAS LabPro software version 5.2.07 (Avisoft Bioacoustics, Berlin, Germany). Spectrograms were measured at 512 FFT length, 100% frame, using a Hamming window. All the vocal elements were identified and manually marked from the sonograms using the Avisoft SASLabPro cursors. For each element, we measured the peak frequency, peak amplitude, fundamental frequency, start time, end time and duration using SASLabPro automatic spectrogram parameters function. Singing bouts were defined by measuring the silent intervals between vocal elements. Intervals above 1 second separated elements into consecutive bouts. Bout duration was calculated by subtracting start time of the first bout element from the end time of the last bout element. To standardize for differences in song length, we converted the time passed from the beginning of the song for each bout element into a percentage of the total song length. This percentage was specified as an explanatory variable in subsequent models.

Amplitude measurements are sensitive to distance and to body orientation of an animal relative to the microphone^45^. During singing, male hyraxes stay at a single location, usually on the top of a raised rock or a tree branch, and keep their position until singing is complete. Changes in body orientation during singing are rare and mostly a result of a distraction, in most cases causing the termination of a singing session. Disturbed songs or interrupted songs were not used in the analysis.

### Bout complexity analysis

Male hyrax songs have been shown to contain a syntactic structure^21^. The relative rate of transitions between different vocal elements can be expressed as a 5 × 5 Markov transition matrix (wail, chuck and snort, with the addition of “bout start” and “bout end” markers), and we used the non-uniformity of this transition matrix to quantify syntactic diversity^46^. We calculated syntax diversity using the weighted sum of the entropies of the transition matrix probabilities, which was shown by Shannon, *et al*.^47^ to be an estimate of the entropy rate of an ergodic Markov chain^48^. We set the “start-end” and “end-start” blocks as zero on all matrices because these transitions are biologically meaningless. The diversity in the rate of element change was calculated as $S=-\sum _{i}{p}_{i}\sum _{j}{p}_{i,j}{\mathrm{log}}_{n}\,{p}_{i,j}$ where *p*_*i*,*j*_ is the probability of transition from element *i* to element *j*, taken from *n* possible elements (*n* = 5), and *p*_*i*_ represents the stationary probability of element *i*. We calculated the diversity of song bouts throughout the length of every song.

### Playback experiments

Playback experiments were performed using a remotely-activated FoxPro Scorpion X1B speaker with a TX200 wireless remote controller (FOXPRO Inc., Lewistown, PA, USA), following our previously published protocols^20, 27^. The speaker was placed before dawn in one of 10 concealed spots in an area frequently visited by focal hyraxes. The speaker was activated once we had observed and positively identified at least one stationary male hyrax or a group of at least four unidentified hyraxes, within a 20–30 m radius. If natural hyrax vocalisation had been heard, the playback initiation was postponed for at least 5 min. The mean ± SE length of songs used for playback experiments was 103 ± 30 sec, as this is a common duration for hyrax singing^20^. The songs were played according to their original duration, with no repetition/looping in any single playback trial. The amplitude of playbacks was calibrated by preliminary trials to match the normal level of hyrax singing (about 80 dB)^36^. We restricted the number of playbacks to two per day/per site in order to minimize disturbance to hyrax daily routine and natural vocal interactions, to prevent hyraxes from becoming accustomed to the speaker location and to avoid desensitization. To minimize any specific site effects, the receivers for playback trials were selected from social groups located throughout the research area. To eliminate receiver familiarity with the singer, we used songs recorded several years previously and/or at a distance of at least 3 km from the playback sites. All behavioural responses of the individuals visible within the 30 m radius of the speaker during playbacks were noted. We considered a singing response as any reply by at least one of the present males within 2.5 min from the end of playback. The 2.5 min threshold was set according to our previously published protocols^20^ and based on the frequency of male hyrax singing^36^. In cases of several males responding, only the first reply was considered for the playback analysis.

We conducted two sets of playback experiments (Appendix 1):

Set A (descending) - In this set, we sought to evaluate the effect of reducing the complexity of song ending on the probability of reply. We selected recordings from our natural song library based on sound quality. Songs were randomly used throughout the playback sessions (the number of repetitions for each song is indicated in Appendix 1). Five natural songs, each performed by a different adult male and with an increased complexity ending (natural ascending - N_ASC_, Appendix 1), were used as the control and as a template for complexity reduction manipulation.

To determine whether decrease in bout length and bout complexity in the last ~20% of the song would also decrease receivers’ reply rate in comparison to the natural control, N_ASC_ templates were digitally manipulated using Avisoft SAS LabPro software version 5.2.07 (Avisoft Bioacoustics, Berlin, Germany). Each song bout was manipulated separately so the original songs bout number was maintained. The shortening of the last bout slightly affected the overall song duration, however, the mean difference in song duration between natural and manipulated songs was negligible (mean ± SE change 6.6 ± 4.8 sec; ~3.5% of the mean song length).

Two synthetic versions of each original recording were created (Figs S1, S2):

Synthetic Monotonic - S_MON_: Singing bouts were shortened by replacing terminal bout elements with background noise from the same recording and the number of transitions between different elements was reduced to cancel out the strong rise in syntactic complexity towards the end of the song (monotonic tracks MON1- MON5, Appendix 1). The manipulated songs had the same bout number and approximately the same duration as the N_ASC_ tracks but lacked the rise in bout duration and complexity towards the end.

Synthetic Descending - S_DSC_: Singing bouts were shortened and the number of element transitions was reduced so that the syntactic complexity would decrease in comparison to the middle of the song (descending tracks DSC1-DSC5, Appendix 1). The resulting songs had the same bout number and approximately same duration as the N_ASC_ tracks but had shorter bouts of reduced complexity towards the end.

The songs in Set A were played 83 times during the 2015–2016 field seasons, at 6 sites, with a mean of 13.8 ± 3.6 playbacks per site.

Set B (ascending) – Using this set we evaluated the effect of increasing the complexity of song ending on the probability of reply. We selected recordings from our natural song library based on sound quality. Songs were randomly used throughout the playback sessions (the number of repetitions for each song is indicated in Appendix 1). The three natural songs, each performed by a different adult male, which had a descending ending (natural descending - N_DSC_, Appendix1), were used as the control and as a template for complexity reduction manipulation.

To determine whether increase in bout length and bout complexity in the last ~20% of the song would also increase receivers’ reply rate in comparison to the natural control, two synthetic versions of each original recording were created (Figs S1, S2):

Synthetic Monotonic - S_MON_: Singing bouts were lengthened and the number of element transitions was increased so that the syntactic complexity would remain similar throughout the whole song (monotonic tracks MON6-MON8, Appendix 1). The added elements originated from the manipulated recording. They were duplicated and arranged while maintaining natural interval between consecutive elements. The resulting songs had the same bout number and approximately same duration as the N_DSC_ tracks but had constant bout length and complexity.

Synthetic Ascending - S_ASC_: Singing bouts were lengthened and the number of element transitions was increased so that the syntactic complexity would increase in comparison to the beginning and the middle of the song (ascending tracks ASC6-ASC8, Appendix 1). The resulting songs had the same bout number and only slighter longer duration than the N_DSC_ tracks but had longer bouts of higher complexity towards the end.

The songs in Set B were played 92 times during the 2015–2016 field seasons, at 11 sites, with a mean ± SE of 8.4 ± 5.3 playbacks per site.

### Statistical analysis

Values of the listed dependent vocal variables (i.e. peak frequency, peak amplitude, fundamental frequency, bout duration, and number of chucks and snorts per bout), measured from male songs, were standardised within song prior to the analyses using the conventional equation (Xi − $\overline{{\rm{X}}}$_song_)/SD_song_ in order to eliminate large-scale differences in values between songs. To test for the effects of male residency (resident or bachelor), singing context (spontaneous or induced) and proportion of song duration (independent variables) on the dependent variables, we used the generalized estimating equation approach (GEE). The variable proportion of song duration scaled the position of each of the focal elements in the song relative to the beginning of the song. GEE are an extension of generalized linear models (GLM) for correlated data (i.e. mixed model), and specifically designed for repeated measures within the same subjects^49^. We set individual as the random effect in all GEE analyses. The Wald χ^2^ statistic was used for testing the significance of each of the effects and their interaction. Multiple comparisons were conducted using the sequential Bonferroni correction. GEE model fitting was done in SPSS (version 23, SPSS Inc.).

## Electronic supplementary material

Fig S1&S2
