# Supplementary material for: The progression pattern of male hyrax songs and the role of climactic ending
Source: Sci Rep. 2017 Jun 5;7:2794. doi: 10.1038/s41598-017-03035-x (PMC5459900; doi:10.1038/s41598-017-03035-x)
Supplement: Supplementary file 1 — Fig S1&S2 [file 41598_2017_3035_MOESM1_ESM.pdf]

The progression pattern of male hyrax songs and the role of climactic ending

Vlad Demartsev[1,2,*], Amiyaal Ilany[3], Arik Kershenbaum[4], Yair Geva[1], Ori Margalit[1], Inbar Schnitzer[3], Adi Barocas[5], Einat Bar-Ziv[6], Lee Koren[3] and Eli Geffen[1]

[1]*Dept. of Zoology, Tel Aviv University, Tel Aviv 69978, Israel.*

[2] *Dept. of Evolutionary Biology and Environmental Studies, University of Zurich, Zurich 8057, Switzerland*

[3]*The Mina and Everard Goodman Faculty of Life Sciences, Bar-Ilan University, Ramat-Gan, 52900 Israel.*

[4] *Dept. of Zoology, University of Cambridge, Cambridge, UK*

[5]*Dept. of Zoology and Physiology and Program in Ecology, University of Wyoming, Laramie, WY 82071, USA.*

[6]*Mitrani Dept. of Desert Ecology, Ben-Gurion University of the Negev, 8499000 Israel*

***Corresponding author**: Vlad Demartsev *Dept. of Zoology, Tel Aviv University, Tel Aviv 69978, Israel. Dept. of Evolutionary Biology and Environmental Studies, University of Zurich, Zurich 8057, Switzerland* E-Mail: demartsev@gmail.com Tel.+41766516340

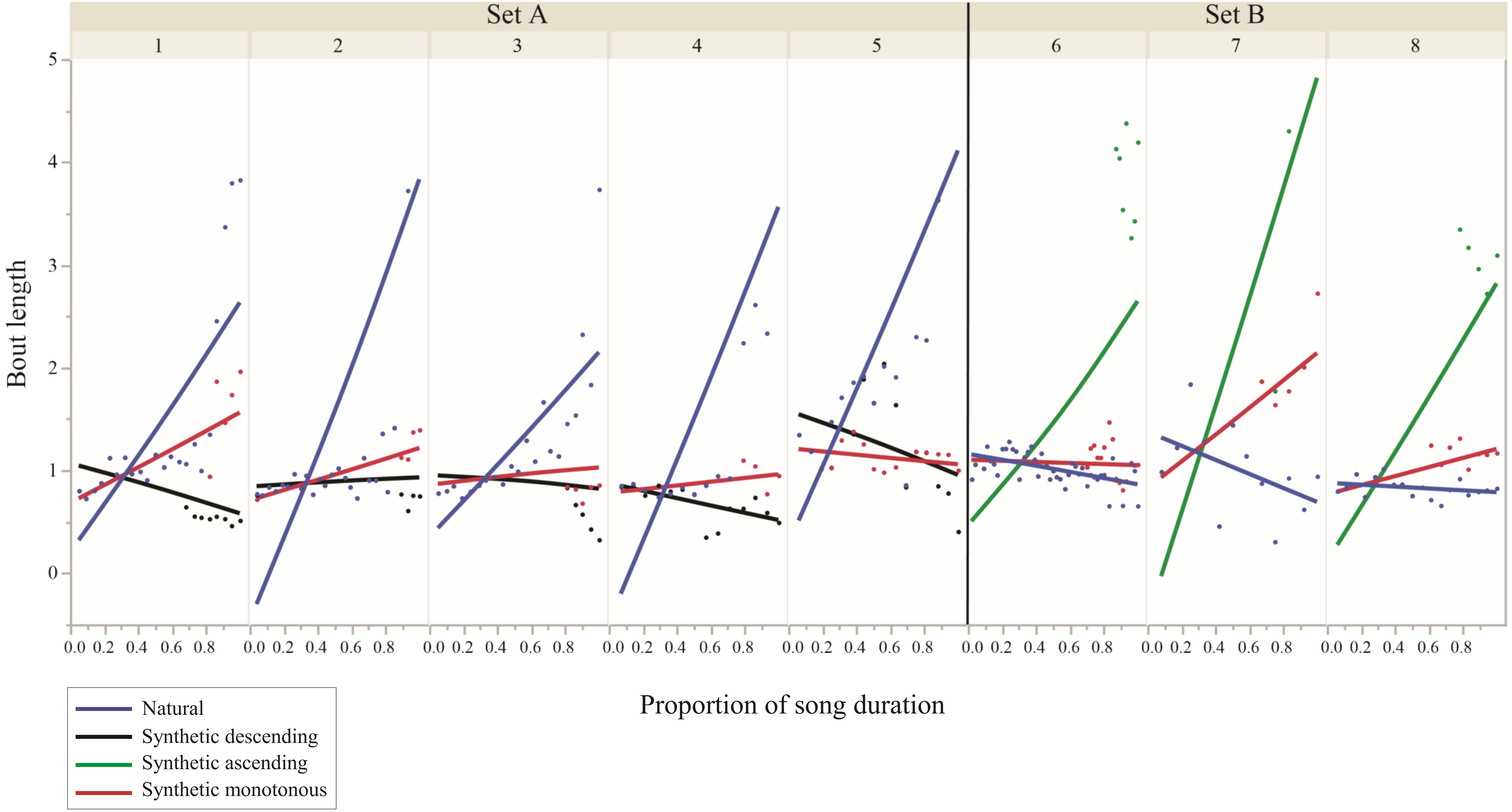

Fig S1: Progression of bout duration (sec) through the proportion of song length for tracks used in playback trials. SetA: Tracks used in trials in which Natural ascending songs (N1-5) were manipulated to have monotonic (MON1-5) or descending (DSC1-5) bout duration. SetB: Tracks used in trials in which Natural descending songs (N6-8) were manipulated to have monotonic (MON6-8) or descending (ASC6-8) bout duration

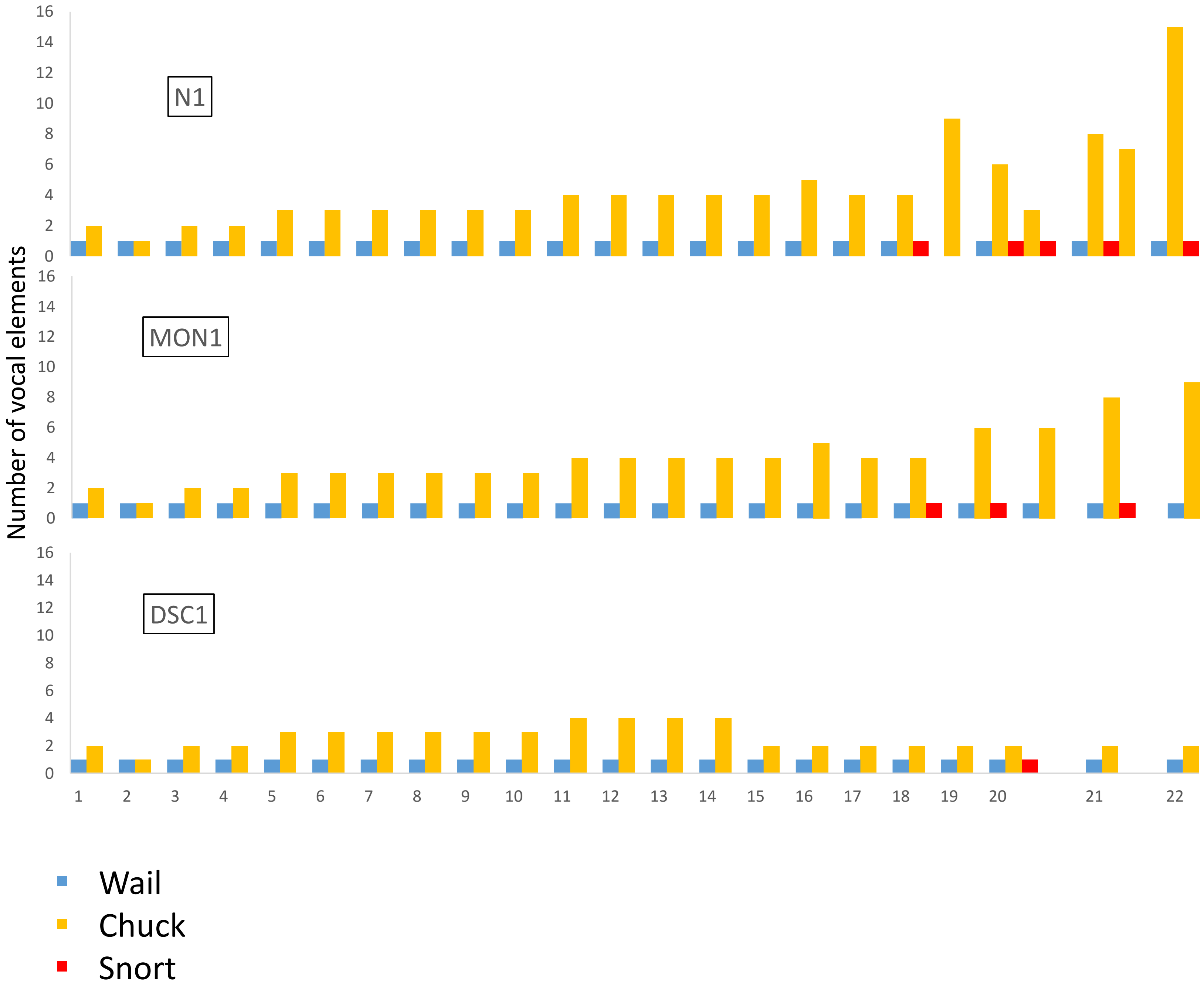

Number of vocal elements
N1
MON1
DSC1
16
14
12
10
8
6
4
2
0
1
2
3
4
5
6
7
8
9
10
11
12
13
14
15
16
17
18
19
20
21
22
Wail
Chuck
Snort

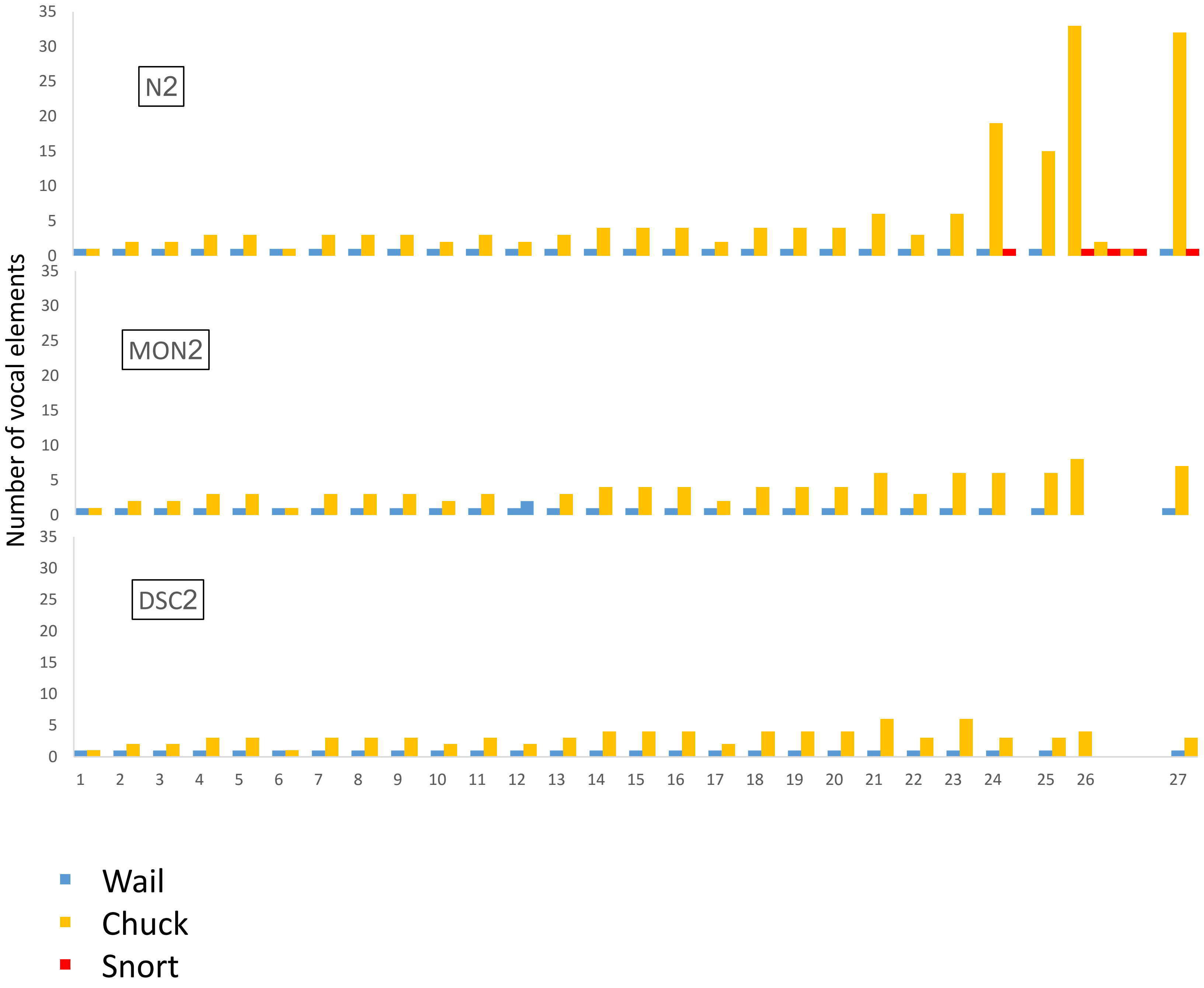

N2
MON2
DSC2
Number of vocal elements
0
5
10
15
20
25
30
35
1
2
3
4
5
6
7
8
9
10
11
12
13
14
15
16
17
18
19
20
21
22
23
24
25
26
27
Wail
Chuck
Snort

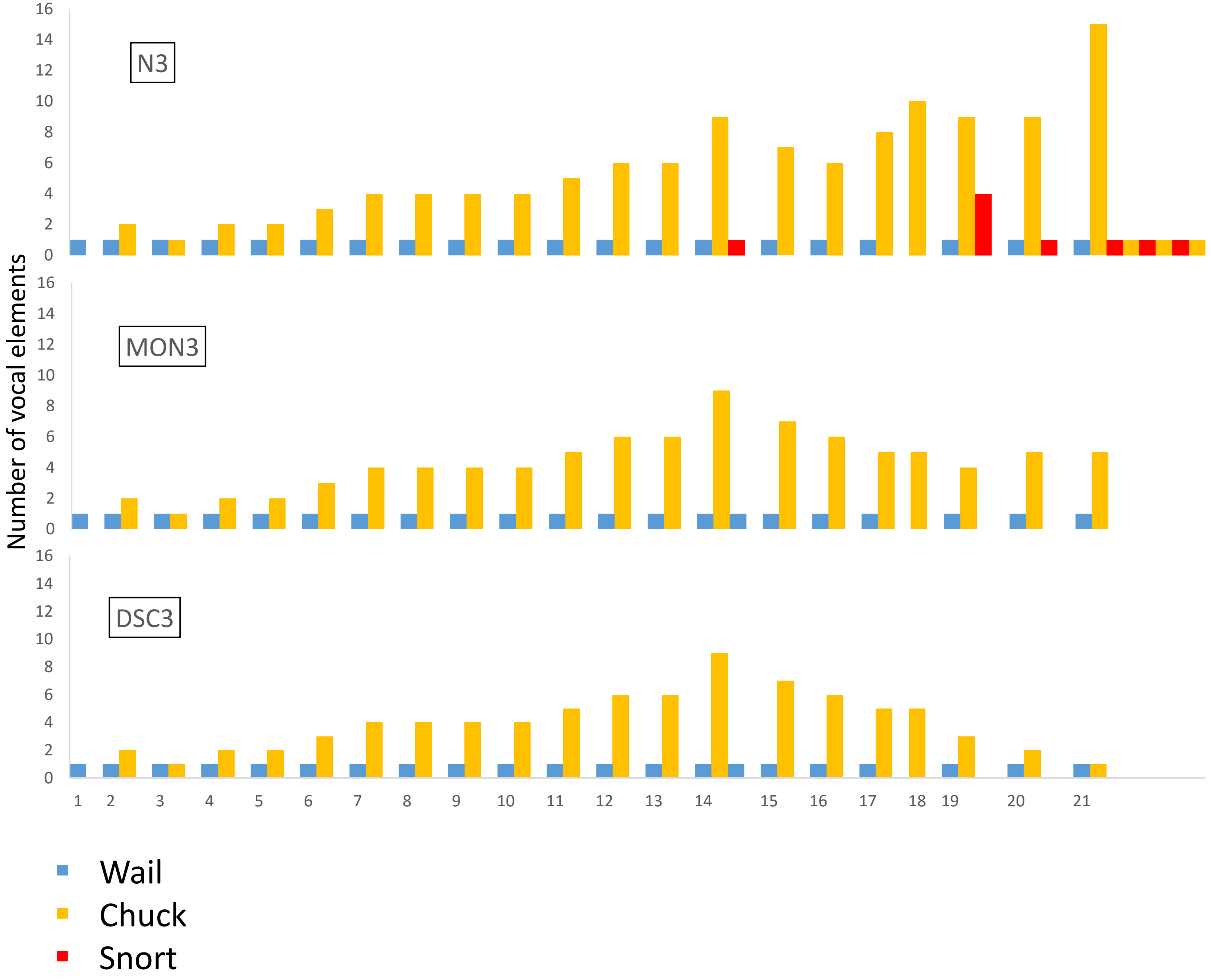

N3
MON3
DSC3
Number of vocal elements
0
2
4
6
8
10
12
14
16
1 2 3 4 5 6 7 8 9 10 11 12 13 14 15 16 17 18 19 20 21
Wail
Chuck
Snort

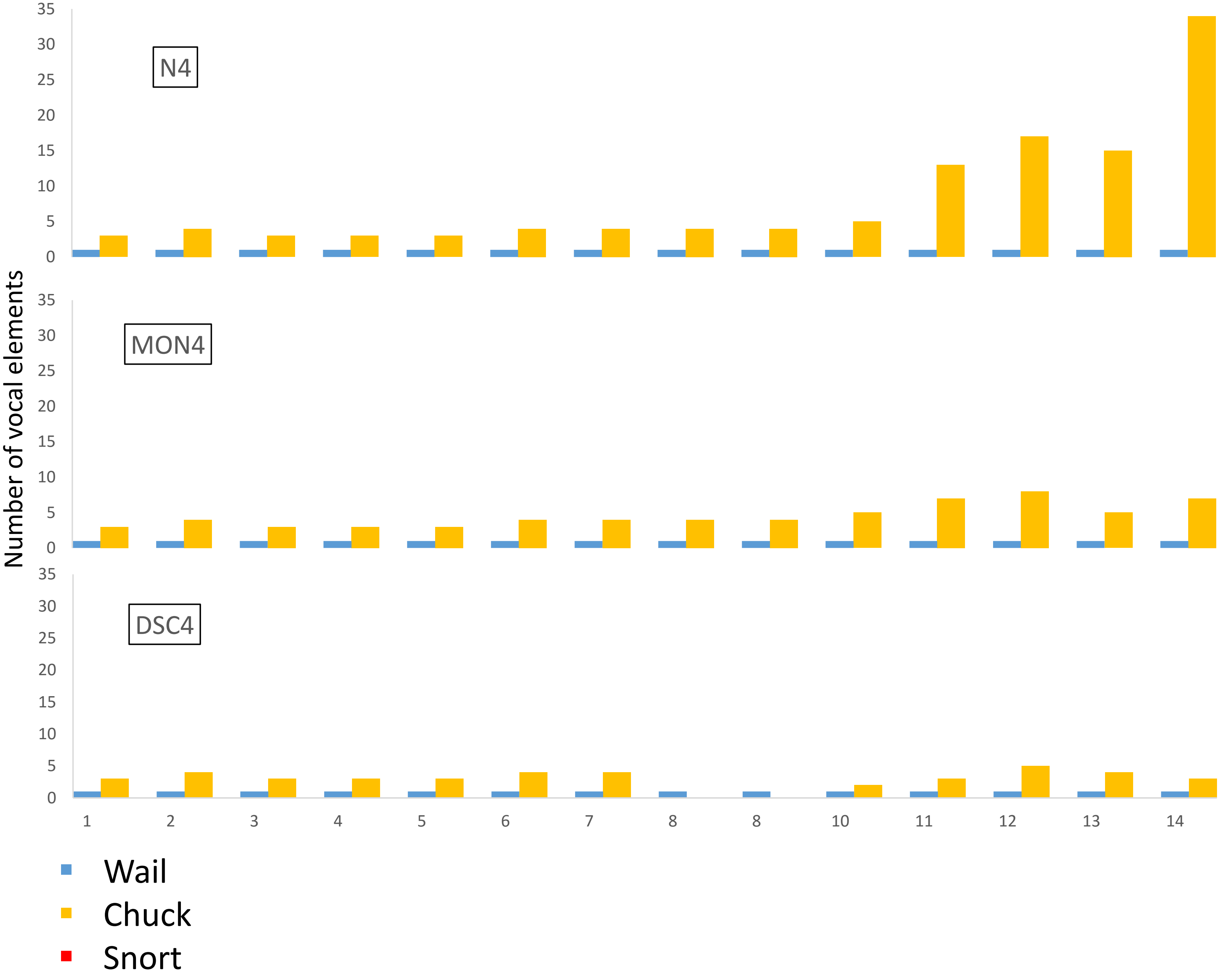
N4
MON4
DSC4
Number of vocal elements
0
5
10
15
20
25
30
35
1
2
3
4
5
6
7
8
8
10
11
12
13
14
Wail
Chuck
Snort

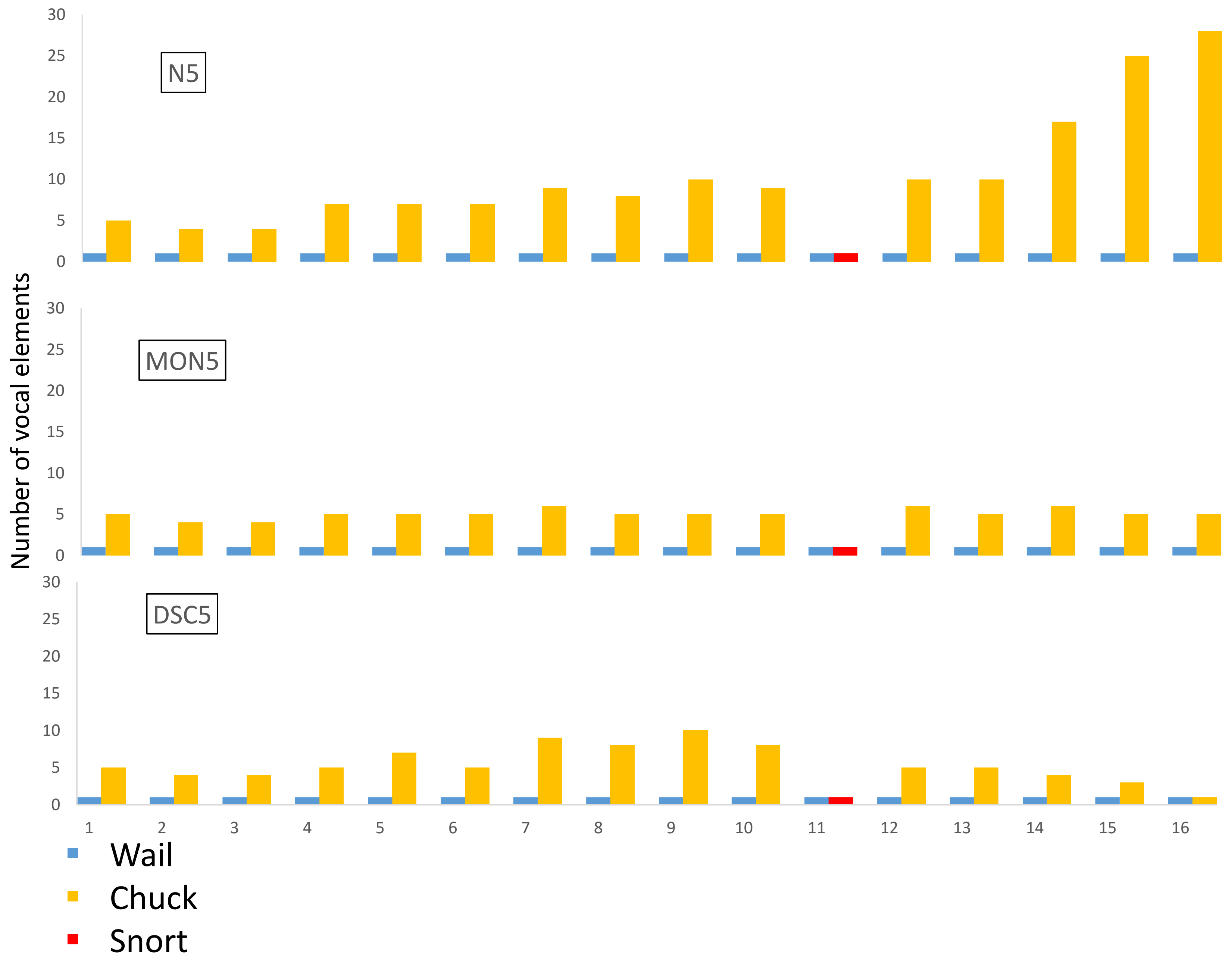

N5
MON5
DSC5
Number of vocal elements
0
5
10
15
20
25
30
1
2
3
4
5
6
7
8
9
10
11
12
13
14
15
16
Wail
Chuck
Snort

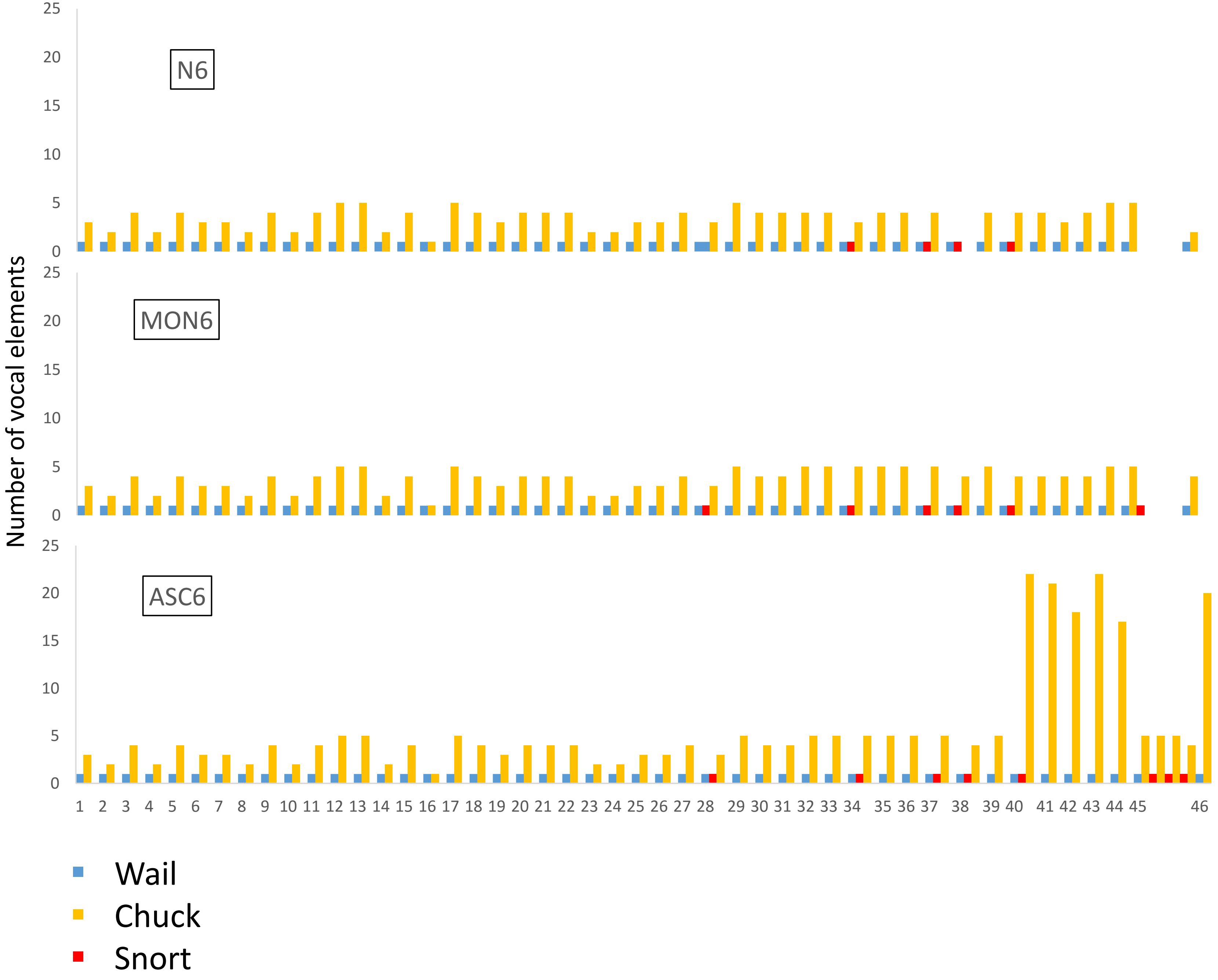

Number of vocal elements
N6
MON6
ASC6
0
5
10
15
20
25
1 2 3 4 5 6 7 8 9 10 11 12 13 14 15 16 17 18 19 20 21 22 23 24 25 26 27 28 29 30 31 32 33 34 35 36 37 38 39 40 41 42 43 44 45 46
Wail
Chuck
Snort

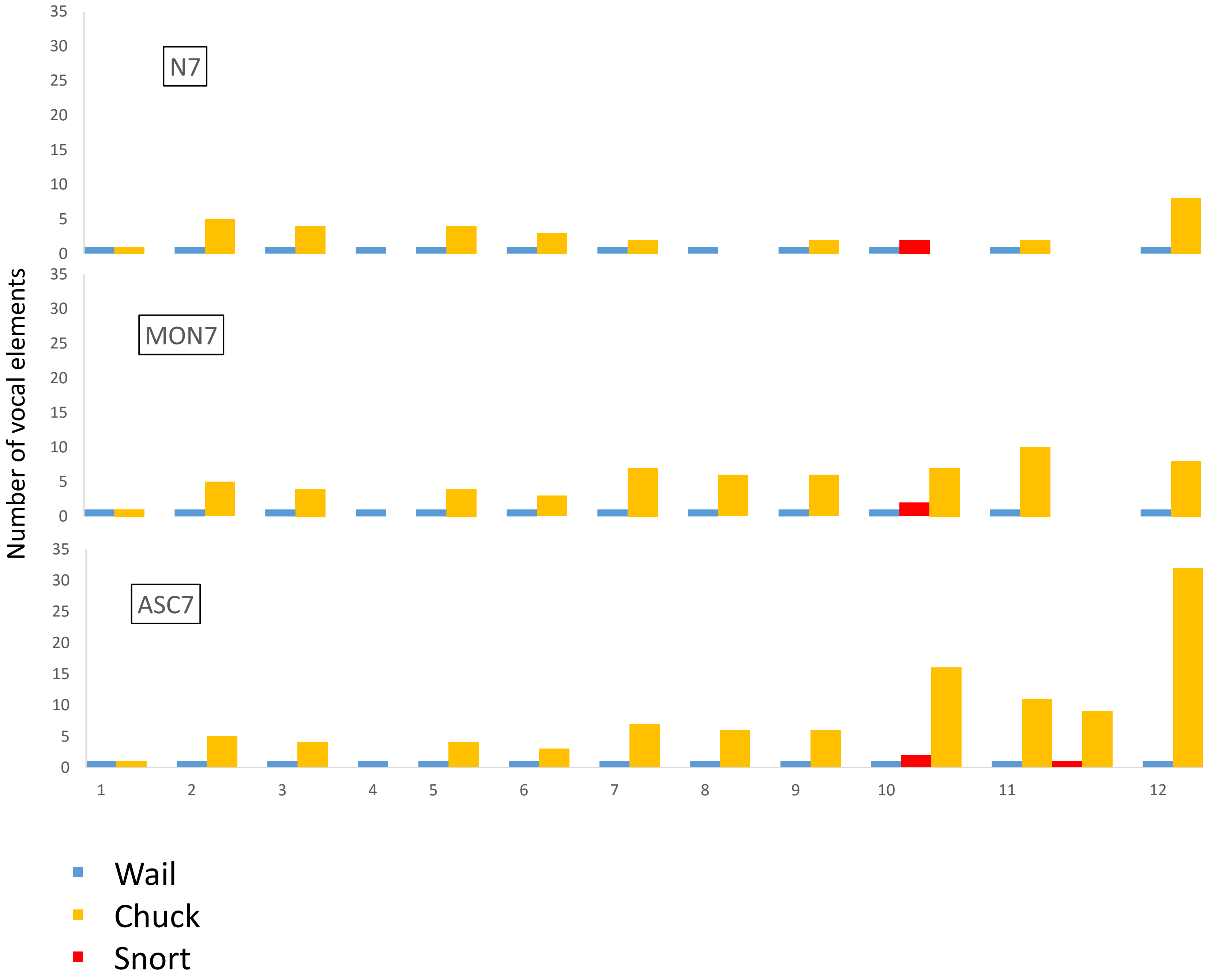
N7
MON7
ASC7
Number of vocal elements
0
5
10
15
20
25
30
35
1
2
3
4
5
6
7
8
9
10
11
12
Wail
Chuck
Snort

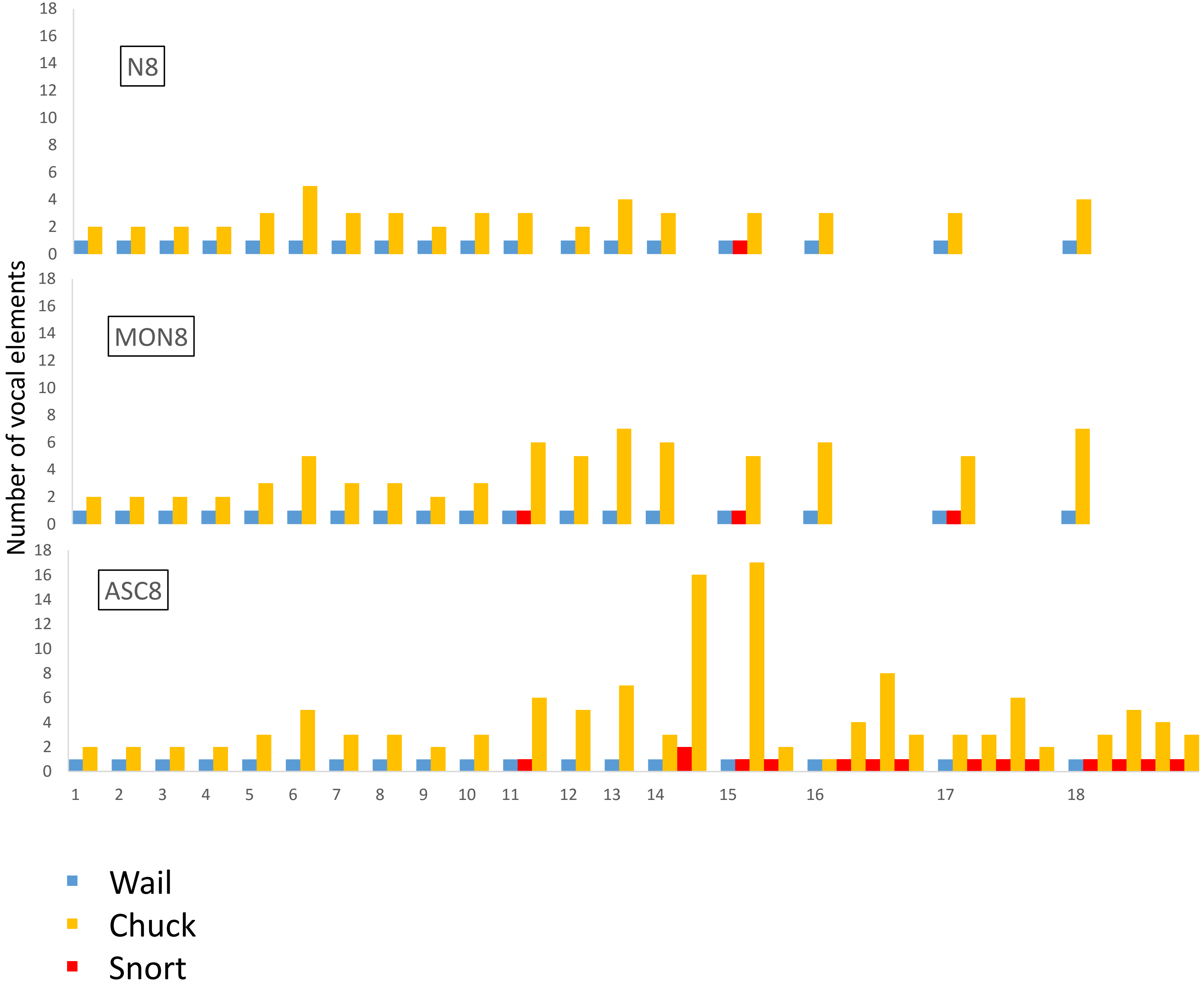
N8
MON8
ASC8
Number of vocal elements
Wail
Chuck
Snort

Fig. S2: Progression plots of vocal element composition over the length of tracks used in playback trials. N= Natural songs with ascending (1-5) or descending (5-8) element progression. MON (1-8) = synthetic songs manipulated to have monotonic element progression. DSC (1-5) = synthetic songs manipulated to have descending element progression. ASC (6-8) = synthetic songs manipulated to have ascending element progression.
